# On the universality of medical device regulations: the case of Benin

**DOI:** 10.1186/s12913-022-08396-2

**Published:** 2022-08-12

**Authors:** A. Maccaro, D. Piaggio, S. Leesurakarn, N. Husen, S. Sekalala, S. Rai, L. Pecchia

**Affiliations:** 1 Applied Biomedical Signal Processing Intelligent eHealth Lab, School of Engineering, University of Warwick, Coventry, CV47AL UK; 2 Institute of Advanced Study, University of Warwick, Coventry, CV47AL UK; 3 The Warwick Interdisciplinary Research Centre for International Development (WICID), University of Warwick, Coventry, CV47AL UK; 4 Nekemte Zonal Health Organization, Oromia, Ethiopia; 5 Università Campus Bio-Medico Di Roma, Via Álvaro del Portillo, 21, 00128 Rome, Italy

**Keywords:** Medical device regulations, Hermeneutic heuristic framework, Low-resource settings, Legal transplantation, Frugal regulation, Bioethics by design

## Abstract

**Background:**

Regulatory frameworks surrounding medical devices (MDs) and medical locations are of utter importance for safeguarding patients and users, and for granting a universal access to healthcare.

Currently, as the main existing regulatory frameworks are drafted by high-income countries, they pretend to be general and applicable globally, but fail to understand particular contexts, specifically those in low-resource settings (LRSs), resulting, therefore, inapplicable. In particular, LRSs present a varied situation, with legal transplants of guidelines from their previous colonial regimes. This apparently theoretical issue, is, effectively, a tangible and rising matter of concern, given the ever-increasing number of MD patent applications per year, as well as the appearance of low- and middle-income countries (LMICs) on the MD market itself.

This article will focus on the European Regulation on MDs 745/2017 and its applicability in LRSs, specifically presenting the case of Benin, a Sub-Saharan African country.

**Methods:**

This work is based on a field study conducted in 2019 in Benin, which is particularly exemplar to show the complexity of the “legal transplantation” concept. A multidisciplinary approach, comprising the standard tools and methods of ethics, law, and biomedical engineering, was used to draft a heuristic hermeneutic framework, and to analyse related bioethical issues concerning Medical Device Regulations (MDRs) in LRSs, the role of Maintenance, and other sociological questions; as well as the rural population’s perception on MDs and health technologies, and the role of ethics in the hospitals of LRSs.

**Results:**

The definition of these themes helped approach the local perspective and define the research questions. Downstream of the analysis of the Medical Devices Regulations, the Maintenance and other bioethical issues in Benin, the heuristic hermeneutic framework was created to guide a shift in the paradigm of law and regulation making, so as to make them more contextualised and inclusive, globally.

**Conclusion:**

This article proposes a framework that will help policymakers take into account the particularism of each context, especially those of the most vulnerable countries, when drafting and issuing regulatory frameworks, promoting an ever-evolving model of universalism.

**Supplementary Information:**

The online version contains supplementary material available at 10.1186/s12913-022-08396-2.

## Background

### Medical device regulations and standards

Medical device regulations (MDRs) are essential for improving public health outcomes and increasing access to safe, efficient, effective and quality medical products [1]. The need for MDRs started surfacing in the early 1960s due to some scandals in the “twin” industry, that of pharma, and was pushed forward by other scandals within the medical device industry (e.g., that of PIP breast implants) [2]. Until the 1970s, the existing regulatory frameworks were based predominantly at the national level, following a prescriptive and subjective approach [3]. Internationally, the situation changed with the Medical Device Amendments (USA, 1976), that aimed to assure the safety and effectiveness of MDs, introducing a risk-based MD classification and establishing the regulatory pathways for new MDs, as well as the post market requirements [4]. These Amendments laid the basis for the European ‘New Approach’, which would constitute the legal foundation of the MD framework of the 1990s, i.e., the Active Implantable Medical Device Directive 90/385/EEC [5] and the Medical Device Directive 93/42/EEC [6], and of the early 2000s Japanese Pharmaceutical Affairs Law.

**Fig. 1:**
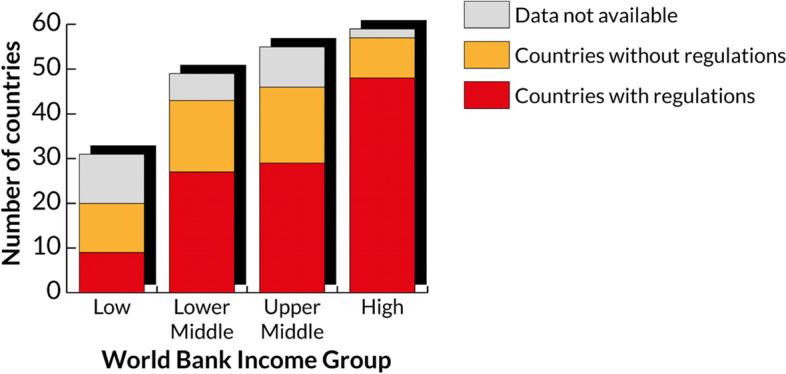
Number of countries with a legal framework for MDs by income group. Adapted from [10]

**Fig. 2:**
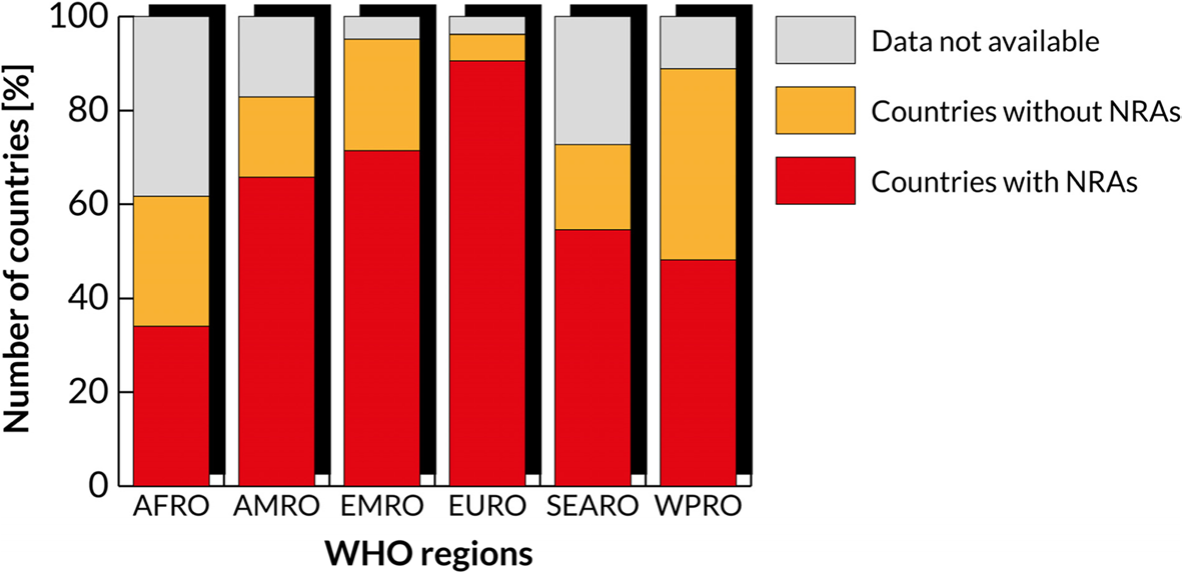
Existence of NRAs by WHO region. Adapted from [10]

Despite an ongoing effort for shifting towards more and more harmonized regulations (e.g., the Global Harmonization Task Force and its successor, i.e., the International Medical Device Regulators Forum^1^), currently, globally, the situation is still fragmented: in fact, the three main existing MDRs are the USA Food and Drug Administration (FDA) MDR, the European MDR 2017/745, and the Japanese Pharmaceutical Affairs Law. All these are currently considered the most relevant ones, because over 75% of the MD market is ruled by these three high-income countries (HICs) [7]. Nonetheless, the MD market shares of these three countries have been diminishing since 2007 (90% of the global MD) [8]. This is due to the emergence of novel fast-growing MD markets, e.g., China, Canada, Brazil, and India [7]. Also Africa, with a compound annual growth rate of about 6% [9], can be included among these novel markets. However, when it comes to regulatory frameworks, there is still a chasm between low- and middle-income countries (LMICs) and HICs. The latter can, in fact, safeguard the safety and efficiency of MDs relying on the presence of very strict MDRs and of National Regulatory Agency (NRAs), when compared to lower-income countries (see Figs. 1 and 2) [10]. Among the World Health Organisation (WHO) regions, the AFRO region is the one with the greatest share of countries without NRAs along with WPRO, while EURO and AMRO are the one with the least share. NRAs are vital because they are in charge of ensuring that the products that are releases for public distribution, including MDs, have been thoroughly reviewed and fulfil the international requirements for safety and quality.

In particular, MDRs focus on three phases of the MD lifecycle, i.e., pre-market, placing on the market, and post-market regulations [10]. As regards pre-market regulations, for many LMICs there is not an official definition of MD, of the MD risk classes nor of their essential principles. Similarly, concerning the placing on the market, several LMICs are lacking an official registration of establishments and listings of MDs and import controls. Lastly, as regards post-market regulations, LMICs have inadequate (if existent) adverse event reporting systems. In respect to this, the Global Atlas of MDs [10] shows that the African Region is the most challenging one regarding the availability of MD regulatory frameworks or data.

A brief summary of the regulatory situation in other world regions follows [11]:

- Asia: Big markets such as China, Russia and Japan have strict MDRs in place; smaller markets in the southeast, belonging to the Association of Southeast Asian Nations, agreed on a MD directive.
- America (not including the USA): In North America, Canada has its own regulation, issued by Health Canada. In South America, there are also strict regulatory systems.
- Africa: Given the wide range of different and complex realities, economic, political, and social instabilities, MDRs in Africa are not well defined, unless the scope of the device is related to the treatment of a specific infectious disease such as malaria, AIDS, and tuberculosis. In such cases, regulations may be present and strengthened by the national regulatory authorities with the aid of help organizations. Further information on the fragmented situation in Africa follows:
  - ◦ Countries with premarket, placing on the market and post market elements: Morocco, Sierra Leone, Burkina Faso, Ghana, Nigeria, Ethiopia, Kenya, and South Africa.
  - ◦ Countries with premarket and placing on the market elements: Algeria, Sudan, Uganda, Rwanda, Tanzania, Zambia, and Cape Verde.
  - ◦ Countries with premarket and post market elements: Egypt and Togo.
  - ◦ Countries with placing on the market elements: Zimbabwe.
  - ◦ Countries without any type of element: Guinea Bissau, Senegal, The Gambia, Mali, Niger, Chad, Libya, Tunisia, Djibouti, Somalia, Ivory Coast, Gabon, Cameroon, Central Republic of Africa, Republic of Congo, Angola, Namibia, Botswana, Lesotho, Eswatini, Burundi, Seychelles, and Madagascar.
  - ◦ Countries with no data available: Mauritania, Guinea-Conakry, Liberia, Benin, South Sudan, Congo Kinshasa, Eritrea, Malawi, Mozambique, Equatorial Guinea, São Tomé and Príncipe.
- Oceania: New Zealand and Australia have solid regulations in place, under MedSafe and the Australian Therapeutic Goods Administration.

#### A focus on West African Economic and Monetary Union (UEMOA)

As this article will focus on the case of Benin, after an overview of the global situation, this section will briefly explain the situation in UEMOA, which Benin is one of the members. The UEMOA is an international organization of 8 West African countries,^2^ created to promote economic integration. This part of Africa was colonized by England, Spain, France, Portugal, and Germany. Therefore, the organization of its healthcare systems is similar to the one established during the colonial period [12]. Beyond economic integration, the harmonization of the regulatory frameworks for drugs and MDs was one of the reasons behind the creation of this union. However, no novel community text regarding this has been published since 2010 [13]. In particular, the latest regulation concerning the harmonization of pharmaceutical regulation within UEMOA dates back to 2005 (Reglement N° 02/2005/CM/UEMOA). However, in 2019, the health ministries of UEMOA reconvened to bring this harmonization forward.^3^

### Aims and scope

This article is innovative in the way it addresses more than one global challenge, i.e., the 3^rd^ United Nations Sustainable Development Goal (Good health and wellbeing), and the 10^th^ (Reducing inequalities), combining multi and inter-disciplinary knowledge and methods (i.e., biomedical engineer, ethics, sociology, law), outclassing the Cartesian fragmentation of knowledge [14]. This will allow the authors to understand and approach the field from the operational perspective of participatory research. In fact, the article offers an interpretative and operative strategy through the proposed hermeneutic heuristic framework. Dissociating from multiculturalism and its concepts of the non-integrability of cultures, such framework builds on the theory of interculture that allows and recognizes the possibility of free cultural and political exchange among countries and cultures without bias,^4^ and proposes new strategies for an inclusive future.

The authors started their research from the surfacing gap between the regulations on MDs and their contextual application in LRSs [15] and addressed this question via 8 field studies from 2016 to 2019 across Sub-Saharan Africa (specifically in Benin, Uganda, Ethiopia and South Africa). Throughout these field studies, the triangulation of qualitative (led by experts in sociology, bioethics and law) and quantitative methods (led by experts in engineering) was used. In fact, the former can complement the latter when used as a crucial initial step to quantitative research and can also be supplementary information to further validate the quantitative results [16, 17]. Such methods include focus groups, content analysis for drafting and validating the questionnaire, pilot tests, semi-structured interviews, medical locations, and MD inspections. The last field study was carried out in November 2019 in Benin, when the multidisciplinary team conducted interviews, surveys, and technical assessment of healthcare locations. The results of the technical part, aimed at assessing the local condition of healthcare settings and MDs, as well as creating a framework for the design of MD resilient to LRSs, have already been published [16, 18]. This article will report the result of the sociological part of the research, aimed at scoping local needs and perspectives in respect to regulations, focusing on the case of Benin. The article will also feature a critical discussion of the results, proposing a framework that enables MDs designers and policy makers to address local needs, and giving a crucial overview from the point of view of international law.

## Methodology

Given the aforementioned purposes, the authors decided to take into consideration and analyse the case of Benin, which can be an excellent case study, as this article will demonstrate. Benin, in fact, does not have a proper MDR in place, and mainly refers to other existing ones, namely the European Regulation on MDs 745/2017, as Europe’s vestiges of colonialism – Benin was a French colony from the late nineteenth century until 1960 – persist in the way that Europe remains a legal point of reference, as well as other regulations based on the provenience of the acquired or donated MDs [19]. Furthermore, Benin is exemplar for evaluating the local and cultural acceptance of medical devices or, more in general, healthcare technologies, as it is considered the birthplace of the Vodoun religion and of a well-spread vivid animistic culture. This peculiar situation inspired the authors to create a multidisciplinary research group, that leverages previous collaboration. In particular, scientific collaboration was instituted among the Beninese Départment du Génie Biomédical (GBM) de l’Ecole Polytechnique d’Abomey-Calavi (EPAC), the Laboratoire de Anthropologie Médicale Appliqué (LAMA) of the same university, the Applied Biomedical Signal Processing and Intelligent e-Health (ABSPIE) Lab and the Warwick Interdisciplinary Research Centre for International Development (WICID) of the Warwick University.

The early phase of this work began in October 2019, when, in light of the upcoming field study in Benin, some of the authors (A.M., D.P., L.P.) met some of the Beninese members of the research group at the ICEHTMC III (Rome, 20–23 Oct 2019). In this occasion, apart from their presentations at the conference,^5^ they had the opportunity to listen WHO’s experts stress the importance of inclusive regulations^6^. Moreover, at the same event, focus groups were held with multidisciplinary experts to highlight the themes of interest that would then be the basis for the semi-structured interviews of the upcoming field study (the full questionnaire can be found in Additional file 1; see Additional file 2 to view summary statistics of some selected interview extracts).

The themes being:

1. MDRs in Low-Resource Setting (LRSs)
2. The role of MD maintenance in LRSs
3. Beninese rural population perception on MDs and health technologies

Based on these themes, a semi-structured interview was drafted, planned, pilot-tested and internally validated via mock interviews and focus groups (Ethical Approval from the University of Warwick Ethical BSREC Committee: REGO-2018–2283), and conducted in the south of Benin, across several cities (Calavi, Cotonou, Zinvié), from November 22 to December 7, 2019. Additionally, we obtained informed consent from every participant prior to data collection. The collected answers were, then, thoroughly analysed from both a bioethical and sociological viewpoint, together with international law experts from the University of Warwick, which led to a discussion on legal transplantation of broad and current interest (presented in Section 4).

### Field study

During the field study in Benin, 18 people (13 males, 5 females), aged 25–60 years, were interviewed in four different hospitals (both public, i.e., CNHU – Centre National Hospitalier Universitaire Hubert K Maga, Hospital d’Abomey-Calavi, and private/religious, as La Croix Zinvié). Among the interviewees there were nurses, medical doctors, administrative staff, biomedical engineering technicians (BMETs) and engineers, members of the Ministry of Health, and scholars considered representatives of the themes object of this research. Possible interviewees were selected if they were directly or indirectly involved with MDs for their work and had at least 1 year of work experience. This means that even if not all the respondents could be defined experts, they can be seen as representatives of people who work daily with such technologies. Out of the 18 interviewees, 5 were from rural/district hospitals and 13 from the public one. The sample was defined using the reasoned choice and snowball techniques. Reasoned choice provides that the sample is chosen in a way that it represents the studied population as accurately as possible. For this reason, as many representatives of the categories involved with MDs as possible were captured. The sample size was determined by the saturation threshold, i.e., the number of interviewees was stopped when no new codes or themes emerged from the interviews, rather, the same ones started recurring [20, 21].

Overall, we believe that this mixed-background population could be a good representative sample of the Beninese population/healthcare workers.

Results of the semi-structured interviews were extracted by a first manual transcription of the audio recording, followed by a coding via NVIVO, which allowed for the thematic grouping of the answers.

## Results

### Results of the interviews

The results are hereby reported by theme.

1. **MDRs.** From the interviews, it emerged that the interviewees are aware of the lack of a Beninese MDR, and that the one of reference is the French one (i.e., the European Regulation on MDs 745/2017), since Benin is a former French colony. As regards the standards, those of reference depend on the type of the device. To this regard, one of the interviewees reported that Benin in 2017 ratified the Medicrime Convention^7^, issued by the European Union in 2011^8^. The signing of this convention is considered to be the early step towards a new regulation, specific for Benin. Alongside this document, some interviewees also referred to others, concerning MDs in Benin, such as the French Public Health Code relating to biomedical equipment and locations (Sixième partie: Etablissements et services de santé (Articles from L6111-1 to L6441-1)), and the Decree of the Minister of Health of 2013 concerning the management of donations.
  Interestingly, according to 94.4% of the interviewees, foreign regulations concerning MDs are not adequate for Benin. In fact, they all claimed to be aware of the need for their contextualization (considering local objective status and subjective perspectives and acceptability), and of the urgency to draft and promote protocols which, while adhering to the reference EU MDR, take into account the particularities of the contexts of applications of such norms.
  When respondents were asked if local BME and technicians share their own concerns over the current status of regulatory frameworks in Benin, they all responded in the affirmative. However, when the question object was shifted to politicians involved in the interview, a criticism was revealed: almost 90% of respondents stated that Beninese politicians have not proposed substantial changes to the existing regulations so far, neither internally nor paying attention to the international community. To this regard, an interviewee explained how the National Strategic Plan relating to MD maintenance, drawn up by the Direction d'Infrastructure d'Equipment et Maintenance (DIEM) of the Beninese Ministry of Public Health, is inspired by European Regulation on MDs 745/2017 and not local needs^9^. One interviewee spoke out their fear hypothesising that: "*the Beninese authorities do not want their own regulation*". This shows the common belief that the politics prefer to adhere to the existing scenarios, that means existing regulations of the HICs, instead of supporting real local improvement.
  Finally, when asked if they perceived the high-resource setting (HRSs)-led drive to identify their own regulations as a form of external imposition, the shared answer was: "*Nemo propheta in patria*", i.e., no man is a prophet in his own land, thus explaining the need to be guided and collaborate in the process of renewal of the regulatory frameworks.
2. **Maintenance**. According to 5.6% of respondents, MD maintenance is underestimated. Related to this, the interviews show the gap between the regulatory dimension and the practical/technological dimension: the administrators are not able to fully understand the challenges that are encountered daily in a hospital in the management of MDs (i.e., the lack of spare parts, the inconsistent electricity, instruction manuals in foreign languages, different temperatures and/or application environments very different from those of construction of an MD, lack of training of technical personnel on new Medical Devices) [16, 18, 19]. This scarce level of communication is also present between the administrative/financial staff of a single hospital and the team of engineers and technicians, who are often not informed of newly purchased Medical Devices or novel incoming donations. This causes great confusions, but, above all, shows how the available MDs do not actually meet the needs of patients/users, and adequate management and use protocols are not followed. In fact, most of the interviewed biomedical engineers and technicians (BMETs) report that even in the event of a failure of a MD, they are often bypassed for self-modification repairs that take place in an amateurish, and often dangerous, way, or for directly contacting the MD manufacturers. With respect to this, an interviewee acknowledges that in the Beninese Code of Public Health, in the Guide to Good Practices of Biomedical Engineering, it reads that no external technician must intervene on the medical devices, if it does not go through the internal Maintenance Service. However, too often the Code is not respected. The reason behind this was given by an interviewee: “*Because people are reluctant to change. The reason is that here they have never seen how the relationship between the maintenance service and the user of the Medical Device should be*". The interviewees reveal how other challenges are a poor consideration of preventive maintenance and quality control of MD, as it often happens that outdated Medical Device is not decommissioned. These issues were also noticed during the technical field studies. Only some hospitals presented a preventative maintenance approach, most of them only put into practice corrective maintenance. Moreover, not all the hospitals had a local team of BME/BMTs, some relied on external “on-call” ones [18, 19].
3. **Perception of MDs.** This section has been included to evaluate not only the popular perception of MDs per se, but also how this can affect local regulations, effectively responding to the needs of the population, in respect of the contextual culture. Ultimately, the results are positive and underline an overall acceptance of the MDs by the Beninese population. Although there is no real hesitation in allowing one’s treatment with MDs, all the interviewees made it clear that the rural population prefers traditional treatments as primary care (e.g., herbal preparations instead of modern drugs). From this, it resulted clear that there is a lack of healthcare staff in charge of a "cultural mediation", i.e., of making sure that each patient has a clear understanding of how the technical tool (i.e., MD), used for their treatment, works. The need for such a role, or for a preventive clarification, which usually falls within the information that the doctor gives the patient during their interaction, becomes crucial in countries where healthcare-patient communication is heavily hindered by illiteracy, language issues (dialects spoken) ​​ and, above all, by the peculiar cultural tradition that characterize medical ethics in such places.

### Towards a hermeneutic heuristic framework

“*To enjoy benefits of scientific progress and its applications*” is a fundamental human right, which belongs to everyone, even more so if it is linked to health and health technologies, as it is stated by the United Nation’s Universal Declaration of Human Rights (art. 27 par. 2) as well as being prioritised by other International Charts (i.e., Article 15 of the International Covenant on Economic, Social and Cultural Rights and the WHO 6 Leadership priorities^10^). In particular, Article 15 of the UNESCO Universal Declaration on Bioethics and Human Rights states: “*Benefits resulting from any scientific research and its applications should be shared with society as a whole and within the international community, in particular with developing countries*”, while, as we have seen, the diffusion of health technologies, in particular MDs, is not universal. Most of the world population does not benefit from the use of MDs: less than 15% of the global population accounts for the use of over 75% of the MDs, suggesting inequitable access to healthcare in favour of higher resource settings [16]. The problem is not only related to the access to the healthcare. Some other principles are involved, i.e., Availability, Adequacy, Accessibility, Affordability and Appropriateness [22], and all of those could impact on the quality, safety and effectiveness of MDs.

The majority of EU/USA citizens accept and trust MDs and are familiar with their use. Conversely, African traditional healthcare is less prone to the use of devices. Moreover, the majority of MDs are designed by and for Europe, USA and Japan, which account for almost the 80% of MD global market [23]. EU, USA, and Japan have homogeneous medical knowledge, clear standards, harmonized regulations and trade-agreements allowing free circulation of MDs among hospitals/countries, while maintaining the same level of safety and efficacy. Consequently, designers and regulators take HRSs hospital infrastructures (e.g., the ones in EU, USA) for granted, completely ignoring the challenges of LRSs.

Some authors have argued that the European Regulation on MDs 745/2017, USA FDA, and Japanese Pharmaceutical Affairs Law are not evidence-based [24], and have historically been written for economic and commercial reasons, and not only to ensure patient safety [25, 26]. Conversely, the African MD manufacturing is not prominent, and African regulations are not homogeneous [27], and often not sufficiently adhered to nor monitored. Unfortunately, USA and EU regulations, despite the allegedly universal criteria, do not consider LRSs local cultures and specific conditions [24]. This evidently intensifies the disparities and inequalities among populations, injuring the human dignity and rights. This reinforces the idea of a western healthcare paternalism that, as an intellectual neo-colonialism, wants to be an imposition of principles and values that are allegedly universal, but result uncontextualized and not aware of objective (climatic and hygienic conditions) and subjective-cultural (the self-representation and identity of different populations).

It could be hypothesised that MDRs rely on some political and economic choices, inspired by Adam Smith’s libertarianism, i.e., the idea of free trade (bound to the idea of liberty, of the “laissez-faire”), which is different from Locke’s liberalism (related to the idea of freedom and to the defence of human rights) [28]. Accordingly, the contained standards and norms will surely have some ethical reasons, but also economical ones, i.e., the idea to easily sell in the common market. This caused competitiveness among “rich countries”, which led to the definition of minimum requirements and criteria impossible to be met by LMICs. As a consequence, most of the MDs designed by HIC are bound to a short-term survival in LRSs,^11^ and MD effectiveness and safety is no longer guaranteed, thus, for a part of the world, principles and rights are not guaranteed either. In this regard, the most compromised principle is that of appropriateness, as MD design is often uncontextualized and this compromises the universalism of norms and standards. On the other side, this attitude results in a defensive closure of the populations in LRSs towards innovative medical healthcare technologies, which are perceived as extraneous.

Regulations should also take into account the fact that different countries have a different historical context, cultures, traditions, perception of healthcare, population priorities, individual perspectives and needs, ethics. However, this should not lead to a relativistic approach that does not allow unitary perspectives, and it can be dangerous, especially for the rights of the most vulnerable people [29]. Conversely, the correct approach should be universalistic, relying on more than one perspective and several principles (other than just economical) and rights that must be considered in their specificity. The aim is, in fact, twofold, i.e., empowering LRSs, and changing perspective towards a reframing of democracy. It is only by expanding the concept of universality, considering simultaneously the particularity of different contexts, with their subjective and objective local aspects, that a full and cosmopolitical citizenship of rights and social equality will be reached.

To address this aim, the authors of this article are working on the definition of an “hermeneutic heuristic framework”. Hermeneutic means interpretative, i.e., able to observe things from a peculiar perspective, that of the interculture, and not of the multiculturalism. Heuristic refers to the underlying methodology: in fact, this framework will be based on the empiricism of inductive reasoning or the “by design” approach that is particularly functional as it allows us to design new work proposals starting from specific. The envisioned framework is interdisciplinary: BME, philosophy, law, ethics are combined to build a strategy of negotiation among disciplines, and among particularism and universalism.

The case study of Benin helped the authors highlight the importance of the particularistic and specific aspects for each context. Benin was exemplary for the deeply rooted traditional religion and culture that has a direct impact on the perception and acceptance of healthcare technologies. Benin is also exemplary because it has no regulation of its own and tries to be aligned with the European one, as it was a former French colony. For all these reasons, Benin is the elective country to represent the main challenges that the authors had identified concerning MDR. This allowed the authors to analyse them with a contextualised approach and propose such framework to overcome the limits of the current situation.

Far from extreme particularism and universalism, and following intercultural mediation, the framework will rely on the moderate or contextualized universalism, as the formulated by Martha Nussbaum and Amartya Sen, who theorized a philosophical and economical model of universalism of rights that takes into account individual “capabilities” (i.e., capability approach). This approach, as suggested by the name, focuses on human capabilities, i.e., what people are actually capable of doing and being, and fights discrimination and unequal treatment, being a fully universal approach [30–32].

According to Nussbaum, this approach can be used to generate political principles by the joint effort of economists, policymakers, political scientists and so on, supported by multinational corporations, global economic policies, agencies agreements, international bodies, and non-governmental organisations. In fact, she strongly believes that each country can pragmatically leverage its capabilities, keeping in mind the SDGs, for a fairer world.

Accordingly, our framework will guide:

- Biomedical engineers and MD designers from EU and Africa in considering key gaps between LRSs and HRSs affecting MDs safety and effectiveness.
- International regulators fostering the harmonization across LMICs and HICs, which will be possible only if there will be a cooperation among Africa- and EU-based experts.
- Policymakers in defining more universal regulations that are not only economy-driven, but prioritise the safeguarding of human rights and dignity.

In fact, the referral to human rights must not be misunderstood as a new type of “transplant”, rather as a tensional reference, an aim to an “universal” that must be taken into consideration in a “situational” way, i.e., by negotiating with the local contexts and their capabilities [33].

**Fig. 3:**
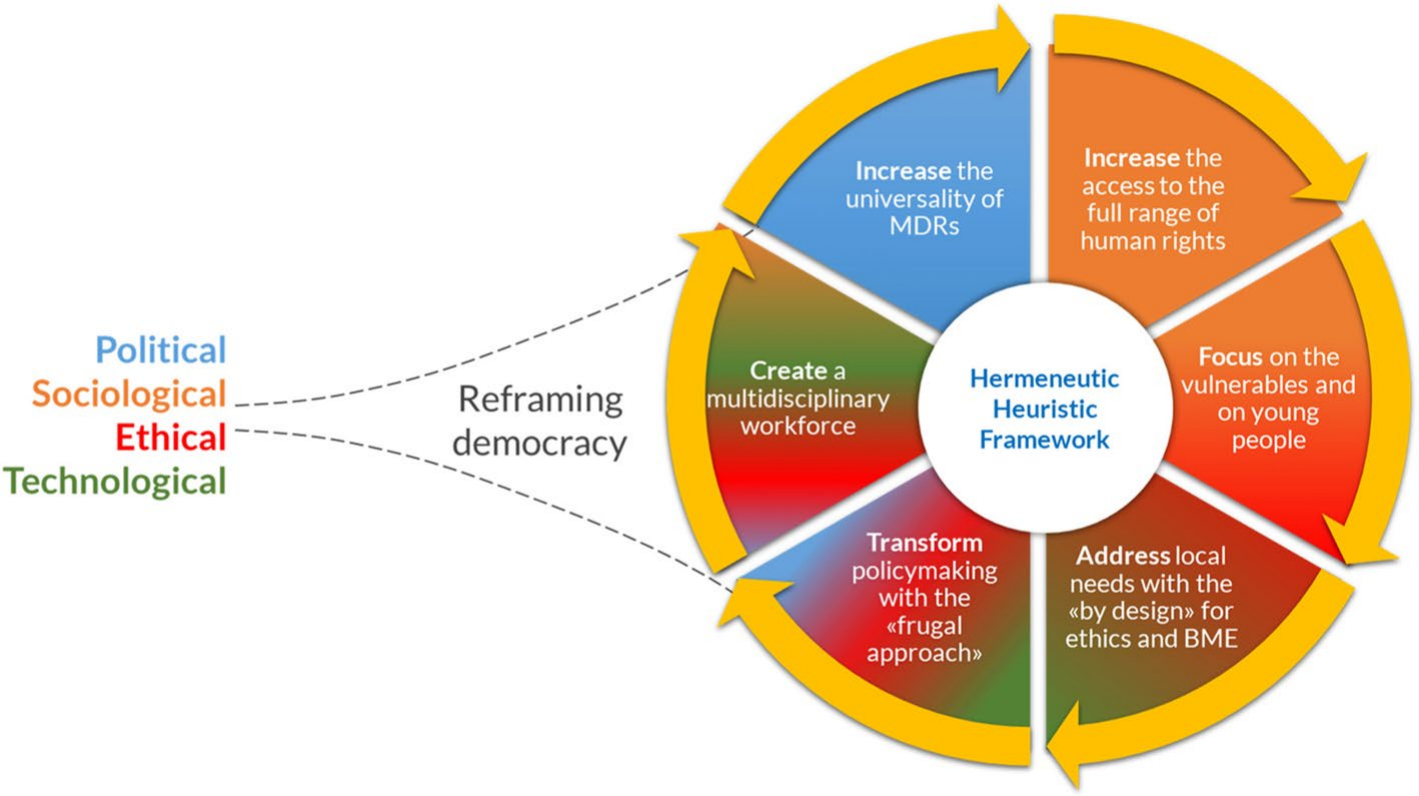
The objectives of the hermeneutic heuristic framework, divided by category, i.e., political, sociological, ethical, and technological

This framework (see Fig. 3) is also crucial to improve communication between scientists and politicians in the public debate. Politicians, in fact, should rely on a more solid scientific culture, as scientists should on a more solid political culture, growing awareness of their social role. In this way, the framework, with its multidisciplinary ramifications, i.e., political, ethical, sociological and technological, could help the increasing of the universality of MDRs, broadening the access to the full range of human rights and, at the same time, with the help of the BME and ethics by design and the frugal regulation approach, address the particularism of each context, respecting and protecting the most vulnerable. The political justice theorist Nancy Fraser argued about the possibility of “reframing justice” [34]. Along the same lines, but starting from a broader multidisciplinary and intercultural perspective, the authors of this hermeneutic heuristic framework think that following this theoretical-practical approach will allow to rethink and, therefore, to redefine and reframe the concept of democracy, including in a transformative way all the cultural specificities and the different social/professional categories mentioned above (legal, political, techno-scientific, etc.) at national and global level.

## Discussions: legal transplantation or frugal regulation?

Section 3.2 laid the basis for our framework. This section will present a put-into-action plan that will follow a heuristic methodology. The first results (engineering related) of this extended study have already been published [16, 18]. Specifically, in [16] a “by design” approach was followed to guide the design of MDs resilient to LRSs. On the same basis, the authors believe that the “by design” approach is also applicable to the sociological and bioethical perspectives. “Ethics by design” is an existing discipline that finds ethical questions based on the characteristics (e.g., tools, algorithms) of novel technologies [35, 36]. However, the interdisciplinary nature of this study led us to introduce “bioethics by design”, i.e., an approach that refers to the concept of life^12^, and, therefore, focuses the design on the needs of the human beings and the multiple questions that emerge from the use of biotechnologies [37, 38]. Although this approach is still being discussed [39], it well suits the present study, because it brings us closer to the problem of technologies (MDs), and their regulations, that should be built following a “bioethics by design” approach, i.e., built on local needs.

But how do laws and regulations become universal and particular at the same time?

Shifting our focus on a legal and policymaking level, we can make a historical reconstruction. With respect to the MDs, it should be clarified that the European Regulatory Frameworks from the 70 s to the 90 s experienced a transition from a subjective and prescriptive approach to a more global, objective one that gradually made producers responsible. The change is witnessed by the fact that also lexically we have recently passed from “directives” to “regulations”, more rigid and uniform than the former. Furthermore, the new regulations are a comprehensive legal instrument, which no longer requires national transposition and is directly applicable and mandatory in each Member State [3].

This is a challenge for Africa, particularly for a countries like Benin, where there are no local regulations, as:

1. There is no drive to draw up and communicate at an international level their own local needs.
2. They often refer to the criteria indicated by the manufacturer, which do not always meet the local conditions.
3. De facto inapplicable regulations are kept in force.

It should be noted that the trend of displacing and imposing a body of norms and laws from its country of origin to another external country is a phenomenon better-known as ‘legal transplantation’ [40].

Legal transplantation theory provides the contexts for cultural difference, the feasibility of legal borrowing, and law and development for the regulatory efficient analysis where regulations/standards are borrowed. The terminology of legal transplantation, also referred to as legal borrowing and legal transfer, is first provided by Alan Watson (1974) [41]. Watson identified transplants as “the moving of a rule or a system of law from one country to another”. Meanwhile, Langer [42] labels transplants as the phenomenon of circulating legal ideas and practices. Geoffrey Samuel [43] terms legal transplants as an approach in comparative law. In understanding the implementation of foreign/international regulations/standards, such as those concerning MDs, therefore, it is useful to examine the legal transplants theory. This will shed light on whether the universal regulations/standards are valid.

The debate on legal transplants is confined to questions as to whether transplantation has been successful. Watson argues that the process of legal transplants in a foreign country is quite simplistic in nature and can be achieved with minimum scholarly expertise [41]. Interestingly, Watson claims that transplanted laws have no links with any socio-cultural influences within a country and that the laws in no way mirror society at large [44]. Conversely, Legrand (1997) argues that legal transplants are impossible and can never be successful [40]. He points out that law is directly derived from a socio-cultural framework, and hence, legal transplantation could vary between cultures and societies. For Legrand the law is heavily influenced by a socio-cultural context and, any interpretations of the law can only be culturally determined, and that a “rule does not have any empirical existence that can be significantly detached from the world of meanings that characterises a legal culture” [45]. Moreover, Teubner argues that the theory put forth by Watson on the distinction between law and society is in direct conflict with social and cultural theories. Several other scholars had contrasting opinions in this regard [46–, 47–50]. Overall, the notion of legal transplants in applying regulations to another country is not valid unless other factors such as culture, social differences, and the country of origin, are also considered by regulators.

Regarding the question concerning the success of legal transplantation, there is no standard of measurement on successful receptions. Although it is difficult to formulate a measure for success and to determine the success or failure of legal transplants, one factor in successful legal transplantation is whether the country is receptive to the maintenance and support of transplanted laws [51]. The process of legal transplantation could be controversial in terms of the responsiveness to legal traditions under new conditions as they need to be “context-specific”. The transplanted laws can only be effective as long as they remain “local” [52].

If legal transplants are to be effective in their design, their implementation has to be considerate of local cultures, religions, socio-economic-political factors as well as geographic differences [48]. As the results vary significantly on a case by case basis regarding which laws can be used for transplants, Kahn-Freund points out that transplants are possible only if the laws can be alienated from their origins [48]. He emphasizes that certain laws are highly rooted in their origins and, thus, cannot be transplanted to other cultures. Moreover, the success of legal borrowing can be determined simply in terms of whether transplanted laws are implemented according to the intentions of the legislators. Therefore, the success of a legal transplant depends on the ability of the host country to adapt foreign regulations and standards to its local conditions and that the transplanted law can serve the objectives of the legislation.

Overall, it is critical to observe and investigate the impact of legal transplantation. The effects of transplants could have unintended consequences on an economy.

In this regard, there is a growing literature on the failure of legal transplants which focuses on why transplanted laws fail in emerging economies [53–55]. One reason for this is that the borrowing of regulations and standards that originate from developed countries may be contradictory to their own systems. For example, they may be due to the political pressures and good will to enforce international standards on all countries. Cranston [56] emphasizes his concerns on blind transplants of the standards, codes and principles of new international financial law or, as he terms it, “neo-colonial domination”. This is important because from another aspect, emerging countries are forced politically to apply the standards of new regulations without consent. Legrand finds that legal transplants should theoretically be aimed at the benefit of society and not for economic advances. Thus, laws favouring developed countries are transplanted into these developing nations to attract foreign direct investments, which boost the economy. Legrand’s contention is that legal transplants must consider local cultural attributes by being aligned to the globalization process, and foreign laws are now gaining more acceptance at the local level [57]. As globalism creates new cultural and social norms, developed countries often tend to take advantage of these norms to create powerful political and economic institutions at the international level [57].

While the debate on legal transplant has found both critics and backers, experts such as Legrand find that legal transplants are an impossible feature in the legal system as they need to cater to the society, they are being transplanted into which may be totally alien to the society the law is being transplanted from. However, other experts such as Watson clearly believe that legal transplants are possible and are not necessarily affected by any impact that socio-cultural factors may play in legal transplants. Legrand argues strongly that legal transplants can cause adverse effects on society and result in undesirable outcomes and, therefore, they must be rejected. However, the available literature on the subject finds that despite its many challenges, there is a role for legal transplants in reforms of the law. Nevertheless, the many challenges facing legal transplants, especially due to globalization, must first be addressed for transplants to become successful.

The fact that the transplant itself refers to the notion of colonialism and the contextual need for a reasoning that still points to the universal (human rights, global health) without being neither generic nor oblivious to particular contexts, invites us refer to Herrick et al. and Tuck et al. [58, 59], who consider decolonization, in this case for global health, as something serious and not metaphorical.

In fact, while these debates on legal transplantation continue, a different ethical-legal tool is proposed for our study, namely that of “frugal” regulations. The definition “frugal” comes from “frugal innovations”, that is the development of appropriate, adaptable, affordable, and accessible goods, services and solutions, especially for the non-affluent customers, in particular in the context of emerging markets [60, 61]. According to Weyrauch and Herstatt “frugal innovation” is defined by three criteria: substantial cost reduction, concentration on core functionalities, and optimised performance level [62]. Precisely for this reason, we think this concept of frugality is applicable to regulations, so that they are sustainable, functional and optimized according to subjective and objective local needs. Furthermore, when referring to “frugal regulations” we mean a provisional structure, designed according to the design of specific needs, and flexible or fluid. This “frugal regulation” is inspired by a learn-by-doing approach, or by the progressive modification and improvement that takes into account the specific context in constant change.

**Fig. 4:**
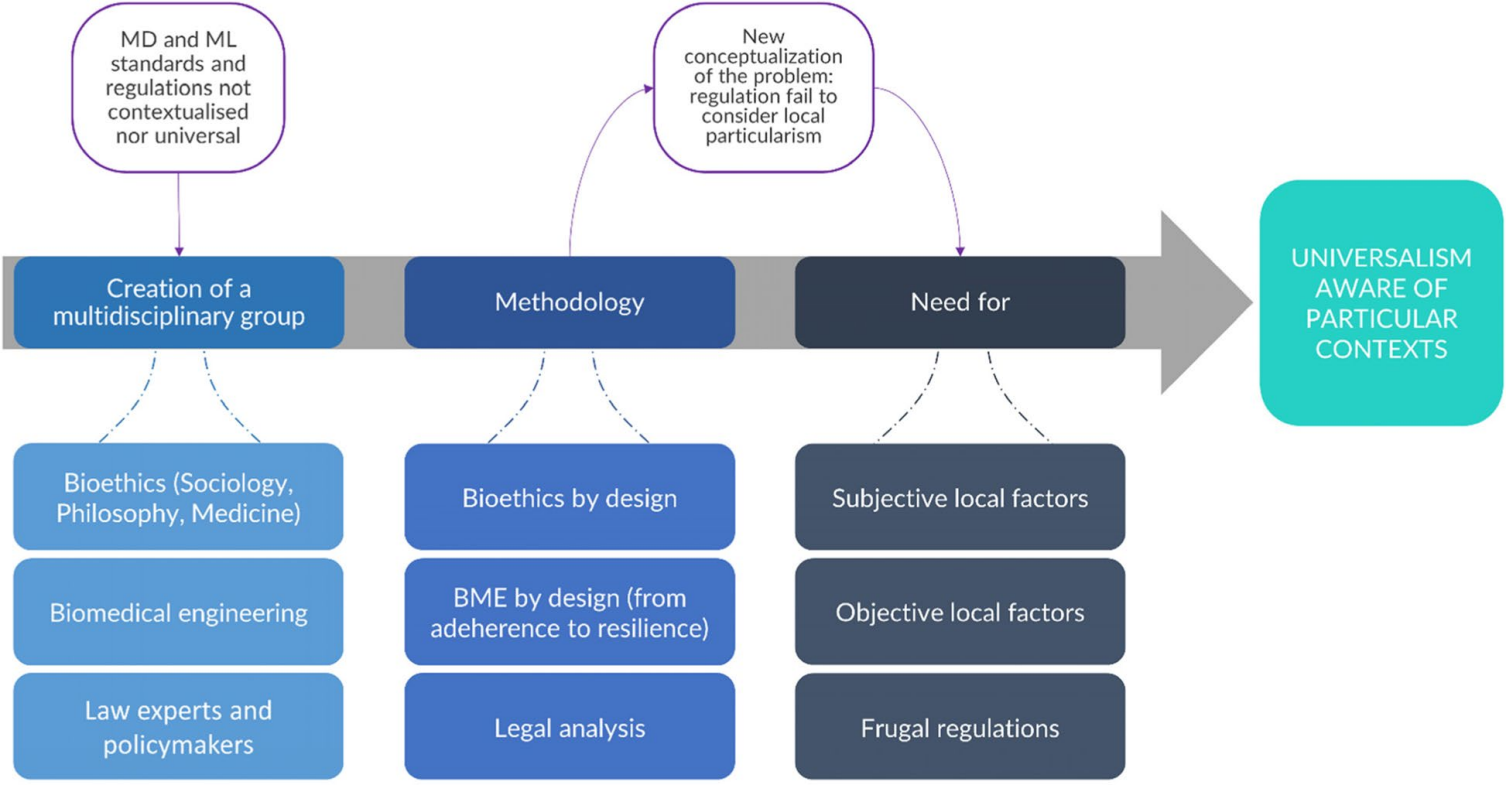
The process behind the creation of the hermeneutic heuristic framework, including methodologies and objectives

“Frugal regulations” represents the last building block of the proposed framework (see Fig. 4) because it envisions a contextualization rather than a self-regulation for each country. This means a negotiation and a by-design adaptation of the commonly stated principles and norms within each culture and local setting, without falling into relativism. Figure 4, in particular, shows how the research question emerged and the steps taken to answer it. The multidisciplinary approach, which allowed the use of different methodologies which shared a bottom up approach. This is particularly evident in the “by-design approach”, which is typical to the domains of ethics and engineering. The research team, therefore, followed an approach that allows the consideration of local factors, objective (i.e., MD location and settings, existing ethical principles, regulation in force, if any) and subjective ones (i.e., perception of healthcare technologies, local traditions of care, traditional culture). This can be considered as a “frugal regulation” approach. With this last piece of the puzzle, it seems possible to propose a new universalism aware of the different specific contexts and,therefore, a flexible regulation that can explain different cultural and traditional contexts without losing its universality and falling into relativism.

The validation of this framework validation is still ongoing, for example:

- at the scientific community level authors have already brought the subject up with the European Alliance of Medical and Biological Engineering and Science (EAMBES) and built a working group that is supporting the WHO, on the definition of a Nomenclature for MDs universal but aware of particular context.
- At the MD design level D.P. is working on several use cases, some of which have been published recently [63, 64], as well as he is spreading the design philosophy presented in [16] in several summer schools.^13^ This design philosophy is aligned with the overarching concepts presented in this paper and its validation will, therefore, be a crucial step for the validation of this framework.
- The International Federation of Medical and Biological Engineering (IFMBE), supported by L.P., launched the first IFMBE Africa biomedical engineering working group. The latter, supported by WHO and African Union,^14^ reunites for the first time 20 African National Scientific societies of BMETs, offering an unprecedented opportunity to dialogue with African experts on MDs and medical locations.
- In 2018, in Ethiopia, L.P. witnessed the birth of the African Community of BME, which aims to bring the African voice to and make it heard at EU level.
- In 2018, L.P., during the European Parliament Interest Group on Biomedical Engineering, organized a meeting to identify possible political actions aiming to reduce the gaps among SSA and European countries, promoting the harmonization of MDs and medical locations regulations. As a result, a task force of the IFMBE wrote down the HTA Guidelines for Medical Devices.
- Networking and collaborations with African scholars in Uganda, Benin, South Africa, Ethiopia, and Mozambique.
- Collaboration with WHO experts (such as Adriana Velasquez and Dr. Sheick Oumar Coulibaly).
- Collaborations with international scientific societies for Health Technology Assessment (IHTA, ISPOR), scientific societies of MD designers (IFMBE, IUPESM, EAMBES) and manufacturers, such as Med Tech.
- Writing scientific publications on the matter and participation in national and international congresses.^15^
- Capacity building in Benin, holding lectures at the University of Abomey-Calavi, raising the students’ awareness of the needs.

## Conclusion

Our field study showed how the local population, and the experts on the subject, are perfectly aware of the MDR problem, and that they require guidance to get out of the impasse, that is, for regulations that are more responsive to local objective and subjective needs. The first results revealed the importance of re-evaluating the role of maintenance, gendered norms, local medical ethics, and prompting politicians to focus more on the local needs rather than on international issues.

The authors followed a bottom-up methodology: starting from “ethics by design”, they proposed a “bioethics by design” that is capable of holding together the interdisciplinary gaze of bioethics and the methodology of design to build an interpretative framework that is effectively able to approach specific contexts and, at the same time, to strive for the universal dimension. Our hermeneutic heuristic framework, in fact, by interpreting the contextual reality in its specific particularity with quantitative and qualitative methods, addresses the political, regulatory, technical, design side. This framework allows LRSs inhabitants themselves to be able to evaluate their own needs, bring them to collective attention, and propose new endogenous strategies for action: in fact, as it suggests to the designers a more attentive approach to the *resilience* (to LRSs) of MDs rather than their *adherence* (to the currently existing standards), at the same time, it suggests to policy makers the use of “frugal regulations”, i.e., rules that are never uniquely defined and always sensitive to change, and to the scientific and cultural progress of a country. This article tried to take stock of and summarise the pieces of a challenging study of which this is but a part.

## Limitations

One might argue that one of the limitations of this study is the limited sample size, which may not be representative of the whole Beninese populations/healthcare workers. However, both the fact that our sample includes people with different cultural and socio-economic backgrounds (different level of experience, education, and positions, etc.) and our use of the aforementioned saturation threshold to capture all the possible themes, make this limitation negligible. Nonetheless, further comparative ethnography studies could focus on other populations of Sub-Saharan Africa to see whether similar beliefs and perceptions are shared within wider communities. 

## Supplementary Information

**Additional file 1.** Medical devices in Africa: ethical aspects and universality of norms.

**Additional file 2.** General information.

## Data Availability

The data generated or analysed during this study are included in this published article [and its supplementary information files].

## Abbreviations

MDs: Medical Devices
BME: Biomedical Engineering
BMETs: Biomedical engineers and technicians
LRSs: Low Resource Settings
LMICs: Low- and Middle-Income Countries
HICs: High Income Countries
SSA: Sub-Saharan Africa
MDRs: Medical device regulations
WHO: World Health Organization
FDA: Food and Drug Administration
NRAs: National Regulatory Agency
UEMOA: West African Economic and Monetary Union
GMB: Départment du Génie Biomédical- Benin
EPAC: Ecole Polytechnique d’Abomey-Calavi
LAMA: Laboratoire de Anthropologie Médicale Appliqué—University of Abomey-Calavi
ABSPIE: Applied Biomedical Signal Processing and Intelligent e-Health Lab – University of Warwick
WICID: Warwick Interdisciplinary Research Centre for International Development – University of Warwick
IHTA: Health Technology Assessment International
ISPOR: International Society for Pharmacoeconomics and Outcomes Research
IFMBE: International Federation of Medical and Biological Engineering
IUPESM: International Union for Physical and Engineering Sciences in Medicine
EAMBES: European Alliance for Medical and Biological Engineering and Sciences

## Footnotes

1 http://www.imdrf.org/

2 Benin, Burkina Faso, Ivory Coast, Guinea-Bissau, Mali, Niger, Senegal, and Togo.

3 http://www.uemoa.int/en/renforcement-du-dispositif-reglementaire-des-produits-et-technologies-de-sante-dans-l-espace-uemoa

4 Multiculturalism, in defending cultural diversity and protecting the rights linked to the identity of each culture, ends up understanding cultures as monolithic identities, making the cultural exchange difficult; Interculturalism assumes that every culture has intrinsic historicity, which opens it up to endogenous modifications and exchanges with others, thus not perverting the sense of the cultural identity of each [15]. Levrau F, Loobuyck P. Introduction: mapping the multiculturalism-interculturalism debate. SpringerOpen; 2018. p. 1-13.

5 Medical devices in LMICs: global gaps, trend and opportunities (L.P.); A device for tracking the photo-pupillary reflex via smartphone in LMICs (D.P.); Medical devices in Africa: ethical aspects and universality of norms (A.M.); Development of IOT-based patient health monitoring and management system (Busola Oronti)

6 How can countries develop Policy and help implement Solutions (Adriana Velazquez, Jitendar Sharma, Tobey Clark, Paolo Lago, Nicolas Pallikarakis, Renato Garcia, Roberto Ayala); The need for integration among BME and MPs, the WHO prospective (Adriana Velazquez Berumen)

7 https://www.coe.int/en/web/medicrime/signatures-/-ratifications

8 https: // www. edqm.eu/en/Medicrime-Convention-Background

9 http://www.pnt-benin.org/wp-content/uploads/2019/02/Benin_PSN_TB_2015-2019.pdf

10 https://www.who.int/about/resources_planning/WHO_GPW12_leadership_priorities.pdf

11 Resolution WHA60.29 about Health Technologies in Sixtieth World Health Assembly, 2007

12 From the Greek βίος, which means “life”.

13 African Biomedical Engineering Consortium Design school (Kampala, Uganda, 2019), Italian National Bioengineering Group (BNG) Annual Bioengineering School (Brixen, Italy, 2022).

14 IFMBE. Kick-off of the First African scientific society of Medical and Biological Engineering 2018 [Available from: http://htad.ifmbe.org/kick-off-of-the-first-african-scientific-society-of-medical-and-biological-engineering/.

15 IUPESM World Congress (Prague, 2018), IV WHO Global Forum on Medical Devices (Visakhapatnam, 2018), ICEHTMC (Rome, 2019), CMBEBIH (Banja Luka, 2019).
