# Supplementary material for: On the universality of medical device regulations: the case of Benin
Source: BMC Health Serv Res. 2022 Aug 12;22:1031. doi: 10.1186/s12913-022-08396-2 (PMC9375389; doi:10.1186/s12913-022-08396-2)
Supplement: Supplementary file 1 — Additional file 1. Medical devices in Africa: ethical aspects and universality of norms. [file 12913_2022_8396_MOESM1_ESM.pdf]

Medical devices in Africa: ethical aspects and universality of norms

This survey aims to identify ethical aspects and norms related to Medical Devices (MD) used by the Beninese government, and the perspective of some Beninese experts of the field. For this reason, the survey will be extended to local biomedical engineers/technicians (BMETs), In order to try to understand, among other things, what the perception of women in the field of biomedical engineering is, considering the traditional gender biases.
This is a multidisciplinary study, which will also focus on the ethical aspects (Ethical Committees, Deontological Codes) for the use of technology in hospitals.
In conclusion, a part of this questionnaire will be focused on the rural population's perception of medical devices, trying to evaluate whether there is reticence about health technologies.
This survey consists of 7 sections, namely Introduction, Personal information, Hospitals ethical situation, Medical Device Regulations, Gender biases, Maintenance of medical devices, Perception of medical devices.
You may be a member of the government or an employee of the hospital (e.g., a nurse, a medical doctor, a BMET) or any person who is familiar with the hospital or expert in MDs.
The answers will be anonymized and used in a research study of the Applied Biomedical Signal Processing and Intelligent eHealth Lab (https://warwick.ac.uk/fac/sci/eng/research/grouplist/biomedicaleng/abspie) at the University of Warwick, led by Dr Leandro Pecchia.
Your answers will not be disclosed and your participation to this study will remain confidential.

Consent

1. Pictures and videos of the interviewee, the medical locations and/or the medical devices may be taken before/during/after the interviews. They will be stored securely and may be used during seminars and as additional materials in journal publications. I hereby authorize the acquisition and treatment of such pictures and videos, as above mentioned.

Mark only one oval.

- Yes
- No
- Other: ______________

2. I hereby authorize, according to the EU regulation 679/2016, the treatment of the transmitted personal data.

Mark only one oval.

- Accept
- Do not accept

Personal Information

This section asks some general questions about your personal information.

3. Date

Example: 7 January 2019

4. District and Country

5. Age

Mark only one oval.

- <20
- 21-30
- 31-40
- 41-50
- 51-60
- 61-70

6. Sex

Mark only one oval.

- Male
- Female
- Prefer not to say

7. The highest grade or level of school/education that you have

Mark only one oval.

- Less than primary school
- Primary school
- Secondary school
- High school or equivalent
- College or University

8. Job

Mark only one oval.

- Minister
- Doctor
- Nurse
- Biomedical engineer/technician
- Other: ______________________

9. How long (years) have you stayed in this job?

Mark only one oval.

- 1-3
- 4-6
- 7-9
- 10 or more

Hospital ethical situation

This section aim to obtain information about ethics in hospitals

10. Hospital/Institution name

____

11. Classification of the Hospital

Mark only one oval.

- Public
- Private
- Religious (specify order)

12. Setting type

Mark only one oval.

- Rural/District Hospital
- Regional/ Provincial Hospital
- National/University Hospital

13. How many patients you are able to treat (beds)?

Mark only one oval.

- ( ) <50
- ( ) 50<X<100
- ( ) >100
- ( ) Do not know
- ( ) We do not have patients
- ( ) Other: ____________________

14. How many doctors are available in your facility?

Mark only one oval.

- ( ) 1-5
- ( ) 5-10
- ( ) 10-15
- ( ) 15-20
- ( ) 20-30
- ( ) 30-50
- ( ) Do not know
- ( ) we do not have doctors
- ( ) Other: ____________________

15. How many of them are women?

Mark only one oval.

- 0-10%
- 10-30%
- 30-50%
- >50%
- Do not know
- We do not have doctors
- Other: ____________________

16. How many nurses are available in your facility?

Mark only one oval.

- 0-5
- 5-10
- 10-20
- 20-30
- >30
- Do not know
- We do not have nurses
- Other: ____________________

17. How many of them are women?

Mark only one oval.

- 0-10%
- 10-20%
- 20-30%
- 30-50%
- >50%
- We do not have nurses
- Other: ______________________

18. How many biomedical engineers/technicians are available in your facility?

____

19. How many of them are women?

____

20. In this Hospital-Institution is there an Ethical Committee?

Mark only one oval.

- Yes
- No
- Other: ______________________

21. In the hospital where you work, do you ask patients to sign a document before a surgery (i.e., informed consent)?

Mark only one oval.

- Yes
- No
- Other: ______________________

22. If the patient is not able to understand the treatment they will undergo, do you provide for the consultation of someone who clarifies all aspects of the treatment?

Mark only one oval.

- Doctors do that
- Nurses do that
- Other: ______________________

23. Has it ever happened that a patient sued the hospital or a doctor for misconduct in the communication of the therapy/surgery?

Mark only one oval.

- yes, often
- yes, sometimes
- yes, rarely
- no, never

24. According to the local culture, whose is the decision to undergo a treatment / intervention / trial?

Tick all that apply.

- [ ] Of the patient
- [ ] Of the family members
- [ ] Of the head of the family
- [ ] Of the husband / father

25. According to the laws of the Republic of Benin, is staff required to comply with a Code of Conduct (Deontologic)?

Mark only one oval.

- ( ) Yes of course, we have a National Conduct Code
- ( ) Yes, each hospital has its own code
- ( ) Not mandatory
- ( ) No, there is no Code of Conduct
- ( ) Other: ______________________

26. Is a Code of Ethics complied with by health personnel?

Mark only one oval.

- ( ) Yes, we have a State Code of Ethics
- ( ) Yes, we have a Code of Ethics of the hospital
- ( ) Not mandatory
- ( ) No

27. How much does the ethical behavior of a doctor / nurse depend on their religion / culture / personal ethics ?

Mark only one oval.

- None
- A little
- Enough
- Very much

28. In your opinion, how good do you consider the role of ethics in the Beninese national healthcare system?

Mark only one oval.

	1	2	3	4	5	
very poor						very good

Medical Devices Regulations

This section aim to clarify the Regulations adopted by Republic of Benin about Medical Devices

29. Are local Medical Device Regulations complied with in Benin?

Mark only one oval.

- Yes
- No

30. If you answered no, to which regulation on medical devices does Benin refer to?

Mark only one oval.

- [ ] American
- [ ] European
- [ ] Asian
- [ ] Do not know
- [ ] Other: ______________________

31. Does Benin have its own medical devices regulation?

Mark only one oval.

- [ ] yes
- [ ] not
- [ ] Do not know

32. In this hospital, do you respect any guideline or regulations on medical devices? if so, which?

____

33. Do Beninese biomedical engineers/technicians perceive European medical device regulations as inadequate to the context?

Mark only one oval.

- [ ] yes
- [ ] not
- [ ] other

34. Have they ever proposed regulatory changes?

Mark only one oval.

- yes
- No

35. Do Beninese politicians consider the European regulations on medical devices inadequate for their country?

Mark only one oval.

- Yes
- No
- Other: ____________________

36. Have they ever proposed regulatory internal changes for their own country?

Mark only one oval.

- yes
- No
- Other: ____________________

37. Have they ever proposed regulatory changes to Europe?

Mark only one oval.

- no, it is not necessary
- no, but it will be necessary
- yes
- other

38. Is there a Biomedical Engineering department in the Ministry of Health of Benin?

Mark only one oval.

- Yes
- No
- Other: ______________________

39. Is there a department of biomedical engineering or clinical engineering or health technology management in this hospital?

Mark only one oval.

- Yes
- No
- Other: ______________________

Gender biases

40. What is the relation between women and science in this country?

____

41. How is a woman involved in biomedical engineering perceived in this country?

Mark only one oval.

- much admired
- appreciated
- normally, as a man
- not very understood
- not very well

42. What is the percentage of female biomedical engineers in this country?

Mark only one oval.

- 0-25%
- 25-50%
- 50-75%
- 75-100%

43. What is the percentage of female biomedical engineers in this hospital?

Mark only one oval.

- 0-25%
- 25-50%
- 50-75%
- 75-100%

44. Who mainly takes care of a medical device maintenance in a hospital?

Mark only one oval.

- Male engineers/technicians
- Female engineers/technicians
- Both
- Other: ____________________

45. In this hospital, who is in charge of medical device maintenance?

____

46. Do you believe that, given the role of the Beninese woman, traditionally linked to home and family maintenance, even medical devices maintenance is considered a "woman's business"?

Mark only one oval.

- Yes, surely
- Yes, could be
- Maybe
- No, absolutely
- other

Maintenance of medical devices

47. How important is medical device maintenance, in your opinion?

Mark only one oval.

- Really Important
- Important
- Not so relevant
- Absolutely not relevant

48. In your opinion, on a scale from 1 (Not degrading) to 5 (Very degrading), how degrading can a job based solely on maintenance be perceived?

Mark only one oval.

	1	2	3	4	5	
Yes, surely						No, absolutely

49. What do you think if the state took actions aimed at fostering the role of women in biomedical engineering?

50. What is the biggest problem of medical device maintenance in Benin?

Mark only one oval.

- ( ) local technicians not trained
- ( ) different language of the instructions
- ( ) no supply chain
- ( ) different setting from the MD country of origin
- ( ) Other: ____________________

Perception of medical devices

This section aim to underline the perception of technology from different respondents

51. How good do you consider medical devices in Benin?

Mark only one oval.

	1	2	3	4	5	
very poor	( )	( )	( )	( )	( )	very good

52. How good do you consider medical devices in this hospital?

Mark only one oval.

	1	2	3	4	5	
very poor	( )	( )	( )	( )	( )	very good

53. How are medical devices perceived by the rural population?

____

54. Do you think that the local population has some reticence about the use of technology and prefers the traditional form of care?

____

55. Who should introduce the rural population to the use of health technologies?

Mark only one oval.

- ethical committee members
- doctors
- nurses
- other
