# Supplementary material for: On the universality of medical device regulations: the case of Benin
Source: BMC Health Serv Res. 2022 Aug 12;22:1031. doi: 10.1186/s12913-022-08396-2 (PMC9375389; doi:10.1186/s12913-022-08396-2)

Supplementary material 2
General information

- Sex

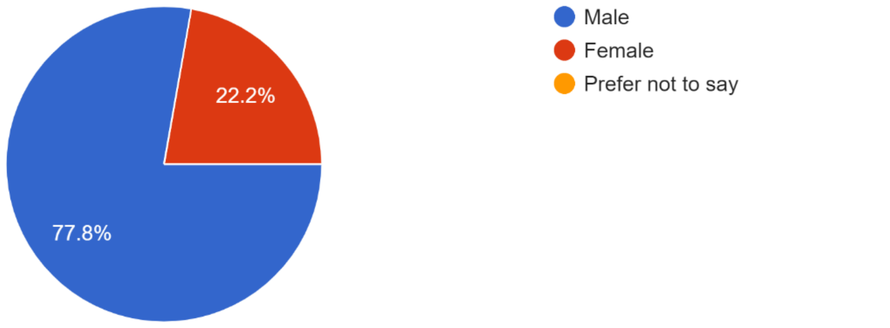

- Job

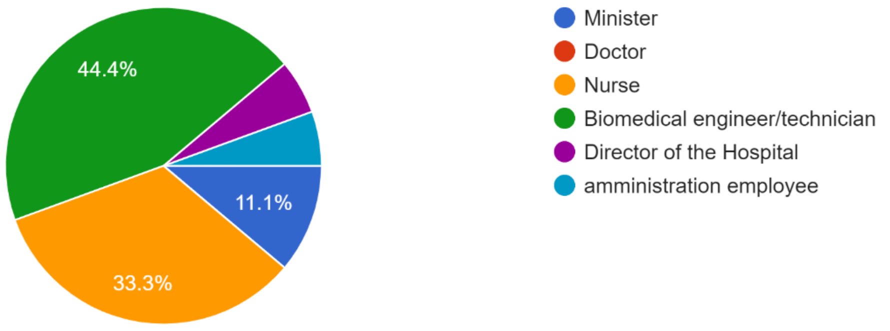

- Classification of hospital

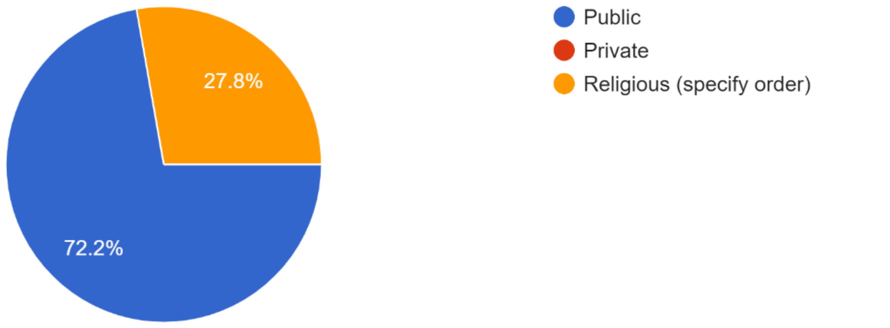

- Setting type

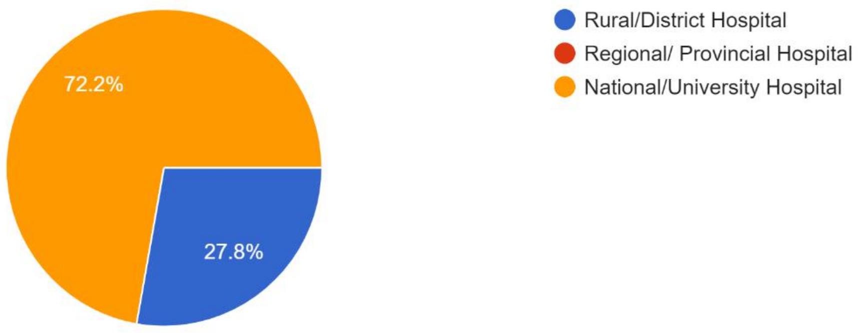

Medical Devices Regulations

- Does Benin have its own regulation for medical devices?

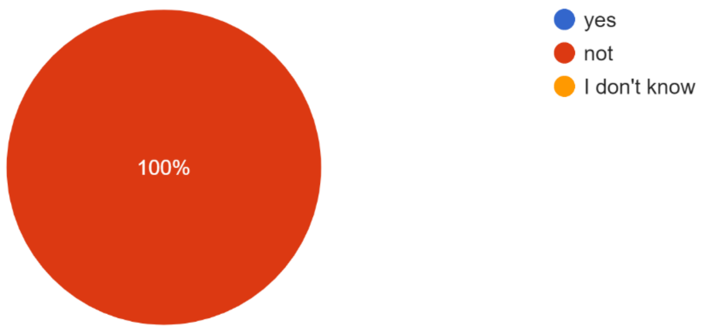

- If you answered no, to which regulation on medical devices does it refer to?

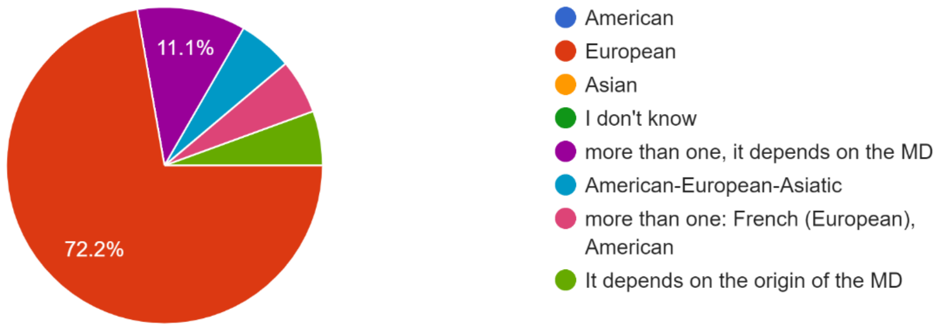

- Do Beninese biomedical engineers/technicians perceive European regulations about medical devices as inadequate to the context?

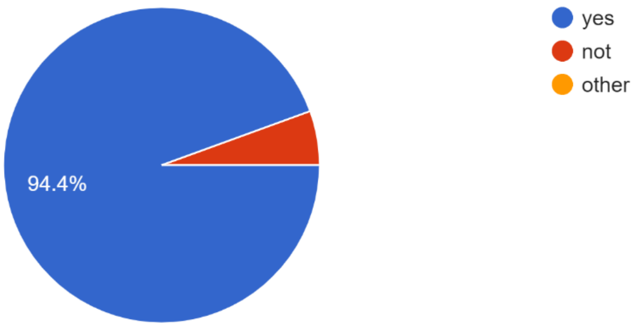

- Do the Beninese politicians consider the European regulations on Medical Devices inadequate for their country?

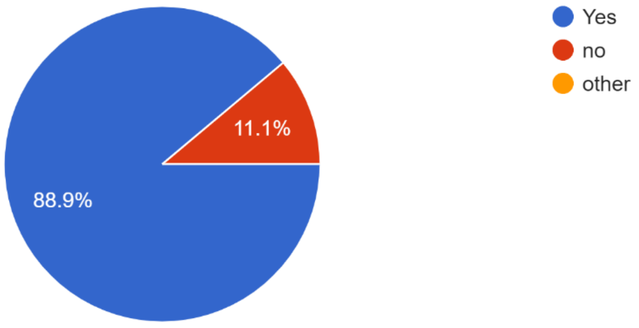

- Have they ever proposed regulatory changes?

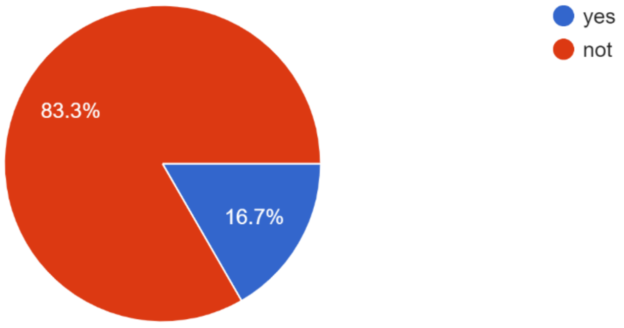

- Have they ever proposed regulatory internal changes for their own country?

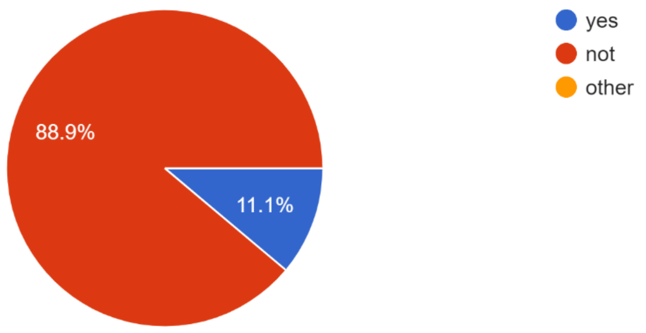

- Have they ever proposed regulatory changes to Europe?

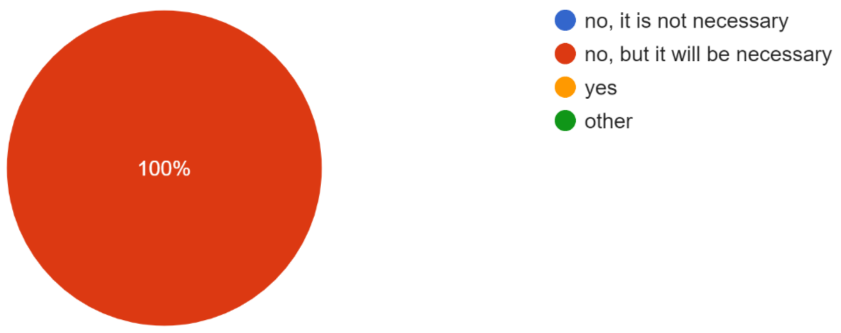

Maintenance

- Is there a Department of Biomedical engineering or clinical engineering or Health Technology Management in this Hospital?

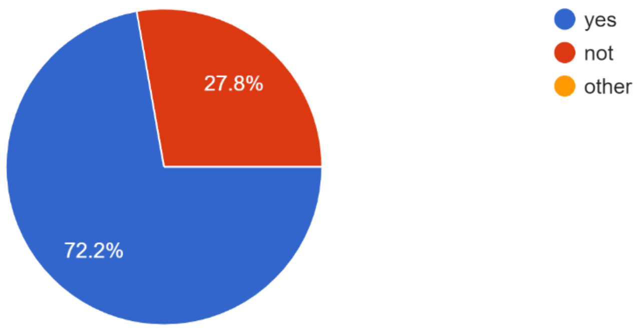

- Who mainly takes care of a medical device maintenance in a hospital?

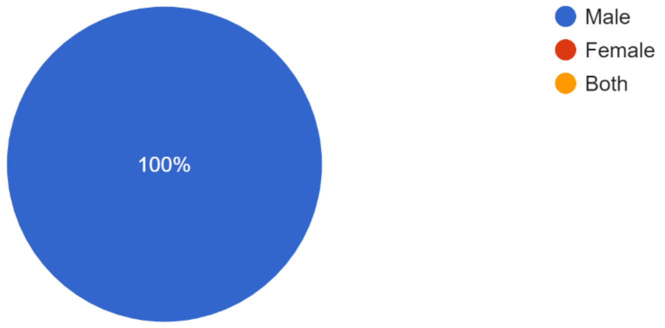

- What is the importance you give to medical device maintenance?

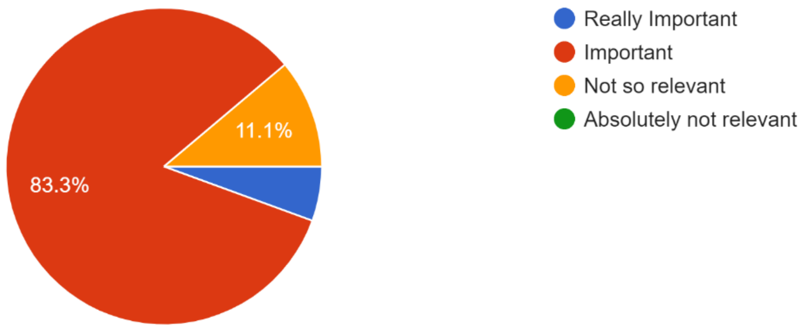

- In your opinion, on a scale from 1 (not degrading) to 5 (very degrading), how degrading can a job based solely on maintenance be perceived?

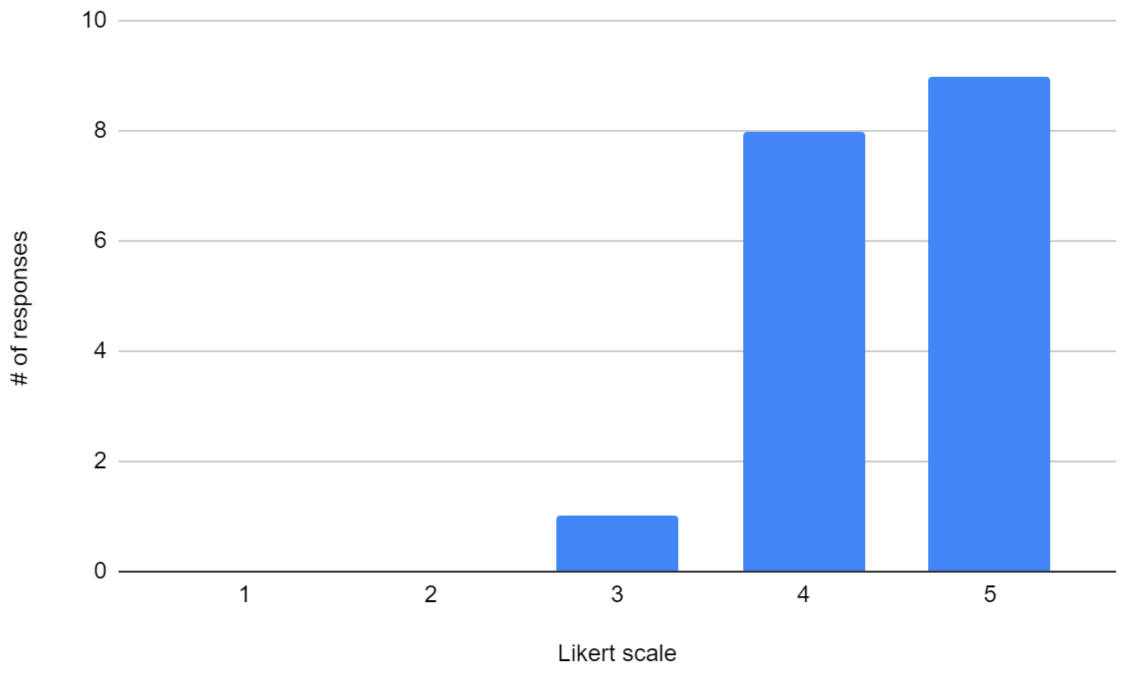

Gender Biases

- How many doctors do you have?

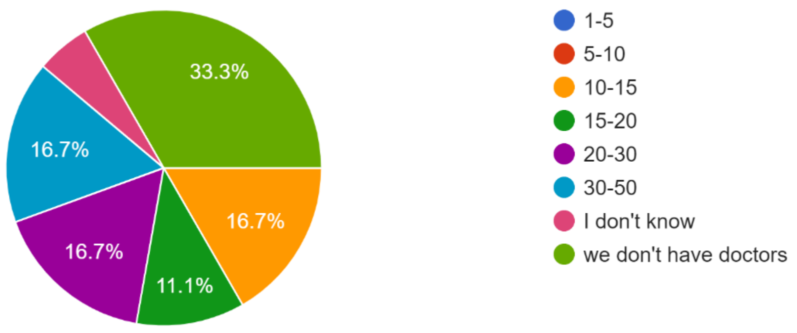

- How many of them are women?

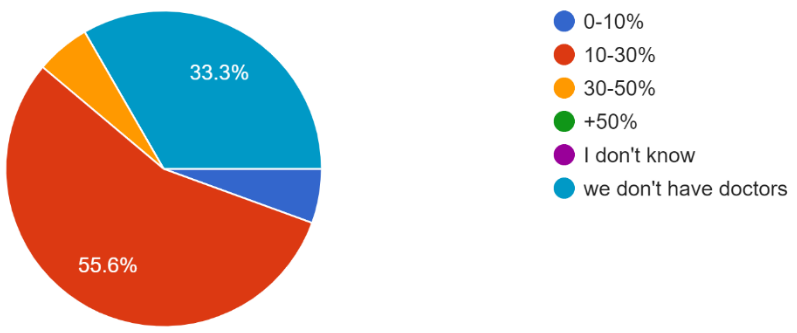

- How many nurse do you have?

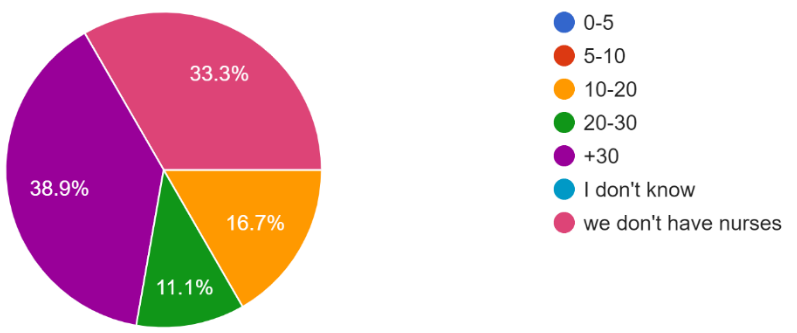

- How many of them are women?

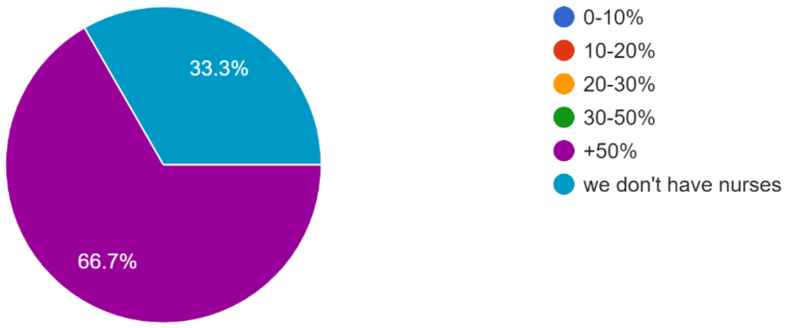

- How many biomedical engineers/technicians are there in your facility?

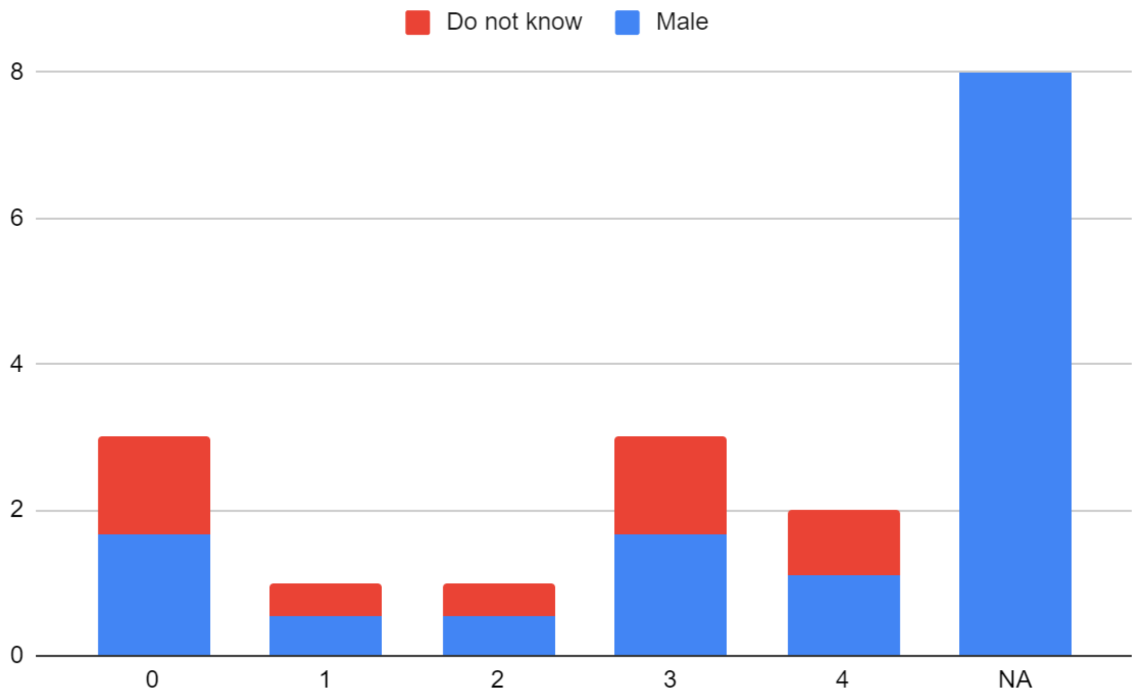

- How is a woman involved in biomedical engineering perceived?

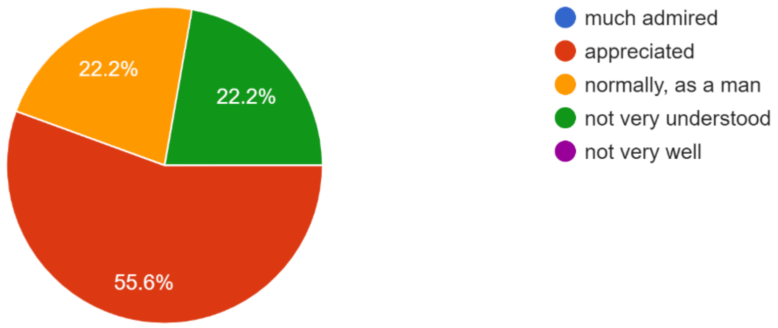

- What is the percentage of female biomedical engineers in this country?

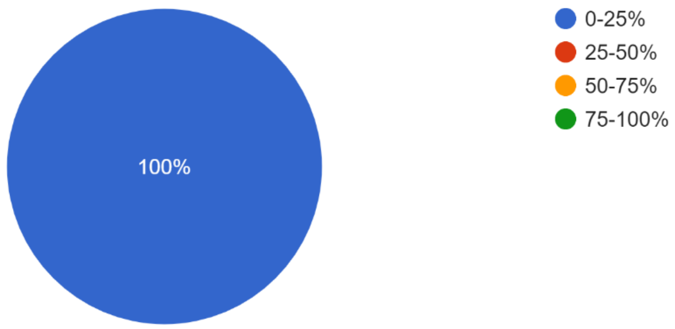

- What is the percentage of female biomedical engineers in this hospital?

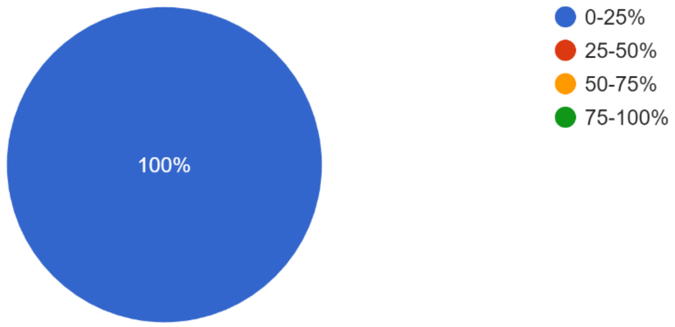

- Do you believe that, given the role of the Beninese woman, traditionally linked to home and family maintenance, even medical devices maintenance is considered a "woman's business"?

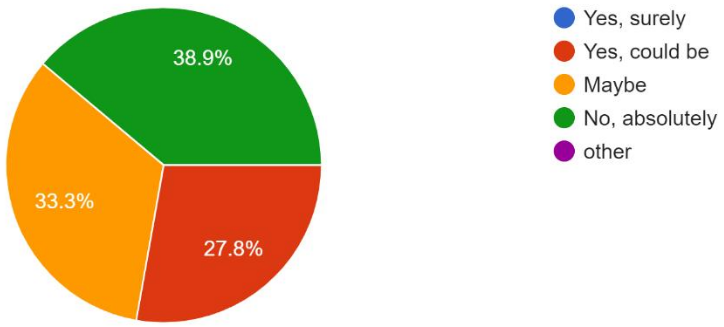

Perception of Medical Devices

- If the patient is not able to understand the treatment they will undergo, do you provide for the consultation of someone who clarifies all aspects of the treatment?

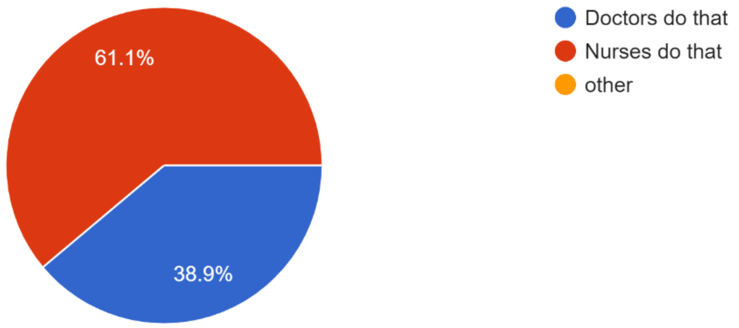

- Has it ever happened that a patient has sued the hospital or a doctor for misconduct in the communication of the therapy/surgery?

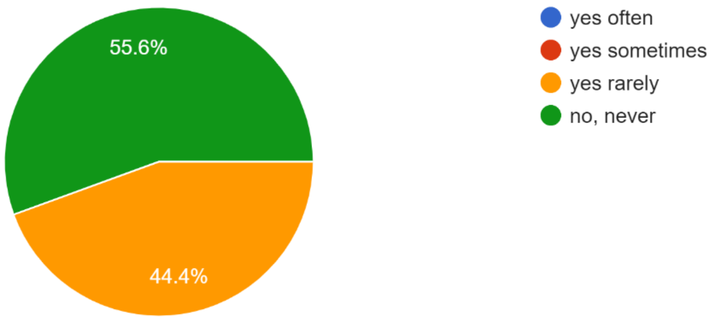

- How are medical devices perceived by the rural population?

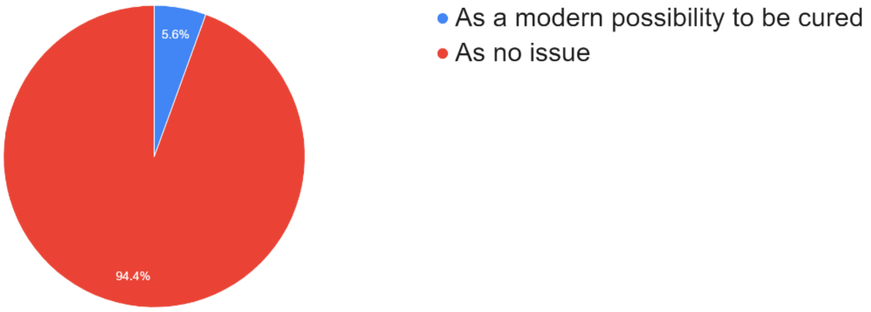

- Do you think that the local population has some reticence in the use of technology and prefers traditional type of care?

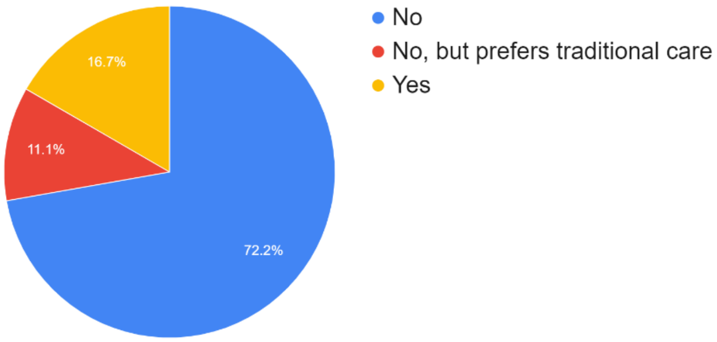

- Who should introduce the rural population to the use of health technologies?

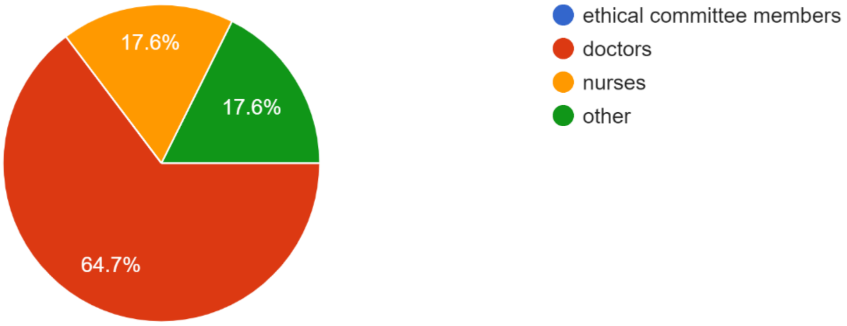

Hospital ethical situation

- According to the laws of the Republic of Benin, is staff required to comply with a Code of Conduct (or Deontological)?

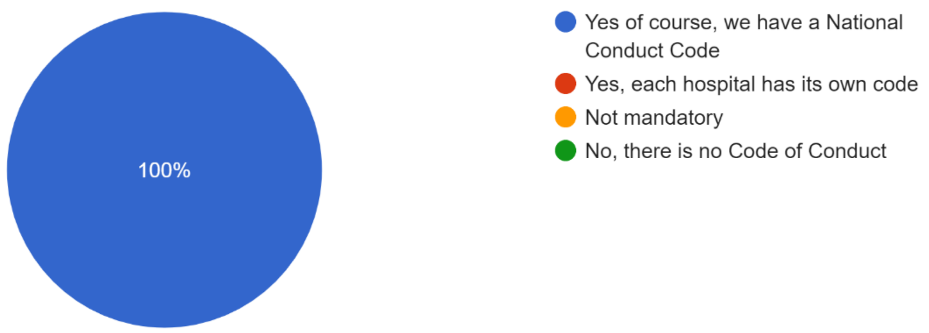

- Is a Code of Ethics complied with by health personnel?

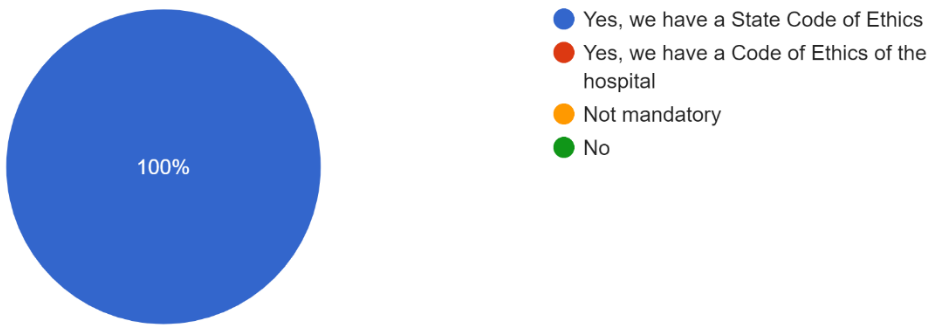

- How much does the ethical behaviour of a doctor/nurse depend on his/her religion/culture/personal ethics?

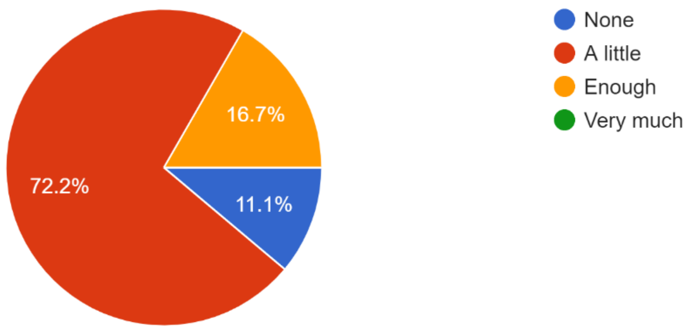

- In this Hospital – Institution do you have an Ethical Committee?

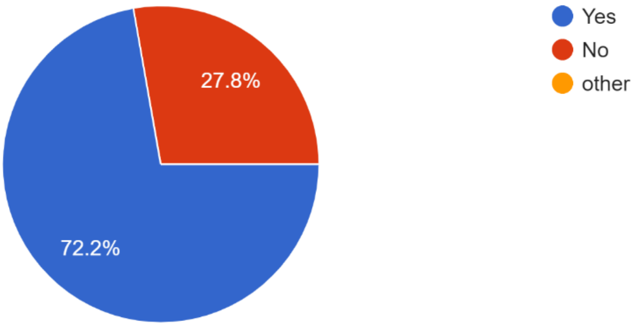

- In the hospital where you work, do you ask patients to sign a document before a surgery (i.e. informed consent)?

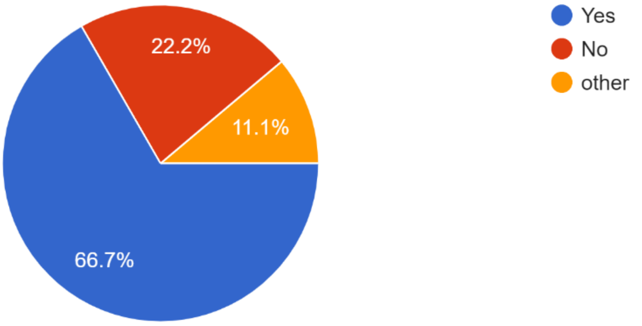

- To whom, according to local culture, in the decision to undergo a treatment/intervention/trial?

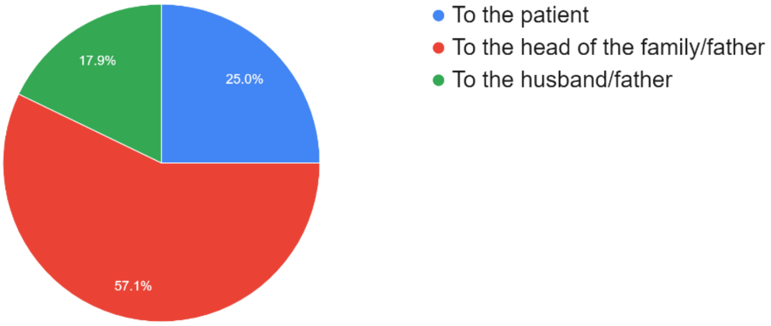

Supplement: Supplementary file 2 — Additional file 2. General information. [file 12913_2022_8396_MOESM2_ESM.pdf]
